# Ginsenoside Rb1 reduces oxidative/carbonyl stress damage and dysfunction of RyR2 in the heart of streptozotocin-induced diabetic rats

**DOI:** 10.1186/s12872-024-04005-8

**Published:** 2024-07-03

**Authors:** Chunpeng feng, Jianping Song, Lan Deng, Jinfeng Zhang, Xinyi Lian, Zhong Zhen, Jinfeng Liu

**Affiliations:** 1 Guang’anmen Hospital of China Academy of Chinese Medical Sciences, No 5. Beixiange Street, Beijing, 100053 China; 2 International Campus, Zhejiang University-University of Edinburgh Institute, Zhejiang University, Haining, China; 3 Jingmen Hospital of Traditional Chinese Medicine, Jingmen, China

**Keywords:** Diabetes, RyR2, Reactive oxygen species, Reactive carbonyl species

## Abstract

**Background:**

Oxidative stress may contribute to cardiac ryanodine receptor (RyR2) dysfunction in diabetic cardiomyopathy. Ginsenoside Rb1 (Rb1) is a major pharmacologically active component of ginseng to treat cardiovascular diseases. Whether Rb1 treat diabetes injured heart remains unknown. This study was to investigate the effect of Rb1 on diabetes injured cardiac muscle tissue and to further investigate its possible molecular pharmacology mechanisms.

**Methods:**

Male Sprague-Dawley rats were injected streptozotocin solution for 2 weeks, followed 6 weeks Rb1 or insulin treatment. The activity of SOD, CAT, Gpx, and the levels of MDA was measured; histological and ultrastructure analyses, RyR2 activity and phosphorylated RyR2(Ser2808) protein expression analyses; and Tunel assay were performed.

**Results:**

There was decreased activity of SOD, CAT, Gpx and increased levels of MDA in the diabetic group from control. Rb1 treatment increased activity of SOD, CAT, Gpx and decreased the levels of MDA as compared with diabetic rats. Neutralizing the RyR2 activity significantly decreased in diabetes from control, and increased in Rb1 treatment group from diabetic group. The expression of phosphorylation of RyR2 Ser2808 was increased in diabetic rats from control, and were attenuated with insulin and Rb1 treatment. Diabetes increased the apoptosis rate, and Rb1 treatment decreased the apoptosis rate. Rb1 and insulin ameliorated myocardial injury in diabetic rats.

**Conclusions:**

These data indicate that Rb1 could be useful for mitigating oxidative damage, reduced phosphorylation of RyR2 Ser2808 and decreased the apoptosis rate of cardiomyocytes in diabetic cardiomyopathy.

## Introduction

Diabetic cardiomyopathy (DCM) is a unique type of heart disease, which is a pathophysiological condition of cardiac structure and function changes in diabetes mellitus without other cardiac risk factors, such as dilated cardiomyopathy, coronary artery disease, hypertension, and other types of heart diseases [1–7]. What we know to date is that chronic hyperglycemia stimulates an increase in an imbalance between oxidative and antioxidative status, and the subsequent augmentation of reactive oxygen species (ROS) and reactive carbonyl species (RCS) in cells and tissues. Type 2 ryanodine receptors (RyR2) are modified after non-enzymatic post-translational modification (PTM) by ROS/RCS, those mechanism should contribute to the heterogeneity in RyR2 activity to lead to characteristic features of the diabetic heart [8–11]. however, pharmacological interventions to prevent or reverse oxidative stress in DCM remain to be developed.

In diabetic myocytes, production of reactive oxygen and nitrogen species (ROS/RNS), and advanced glycation end-product (AGE) is from different metabolic pathways including glycolytic, hexosamine, protein kinase C, polyol and advanced glycation end-product (AGE) pathways [12, 13]. This accumulation of ROS/RNS react on myocytes to increase production of the lipid-derived RCS such as malondialdehyde (MDA), and 4-hydroxy-2-nonenal (4-HNE) [14]. Our previous studies showed that RyR2 represents the most common type of non-enzymatic post-translational modification (PTM) by RCS/ROS contribute to the heterogeneity in RyR2 activity in a rat model of Type 1 diabetes [11]. Oxidative stress has been demonstrated in many studies in the progression of diabetes which plays important role during the occurrence and development of diabetes complications.

We previously demonstrated that RyR2 acquire a gain-of-function phenotype from type 1 diabetic.

rat cardiomyopathy [15]; Increased functional activity of RyR2 should be related to RyR2 phosphorylation status, which could be contribute part of mechanism of various forms of heart failure and DCM. More recently, hyperphosphorylation at a single amino acid residue, Ser-2808, has been proposed to directly disrupt the binding of a 12.6-kDa FK506-binding protein (FKBP12.6) to RyR2, leading to RyR2 dysfunction and dysfunctional Ca2+-signaling pathways in cardiac myocardial tissue [16–18]. The changes in activity of RyR2 could be related to the treatment of DCM.

Inhibition of oxidative and carbonyl stress and phosphorylation of RyR2 have been suggested to deeply evaluate the therapeutical targets in diabetic cardiomyopathy. Exploring the possibility of new use drug therapies has the potential to identify treatments for comorbid conditions that have the added benefit of glycemic control [19–21]. To the best of our knowledge, rare medicines reduce both oxidative stress and dysfunction in RyR2. Ginseng [Panax ginseng C.A.Meyer (Araliaceae)] is a commonly used Chinese medicinal herb to protect cardiovascular disease. Ginsenosides Rb1(Rb1, molecular formula: C54H92O23, MW: 1109.29 g/mol) is a biologically active component of ginseng that is responsible for its pharmacological properties [22–26]. Numerous studies have demonstrated that Rb1 ameliorates diabetic cardiomyopathy and myocardial ischemia by inhibiting RyR2 activity to regulate calcium signaling [27, 28]. However, the underlying mechanisms that confer these effects remain poorly understood. Therefore, Thus, the objectives of the present study were (i) to determine relative levels of oxidative stress and Phospho-RyR2 (Ser2808) from hearts of control and streptozotocin (STZ)-induced diabetic rats with cardiomyopathy, and (ii) to assess efficacies of Rb1 in scavenging oxidative stress and treatments.

to blunt formation of phosphorylation on RyR2.

## Methods

### Chemicals and drugs

**Fig. 1:**
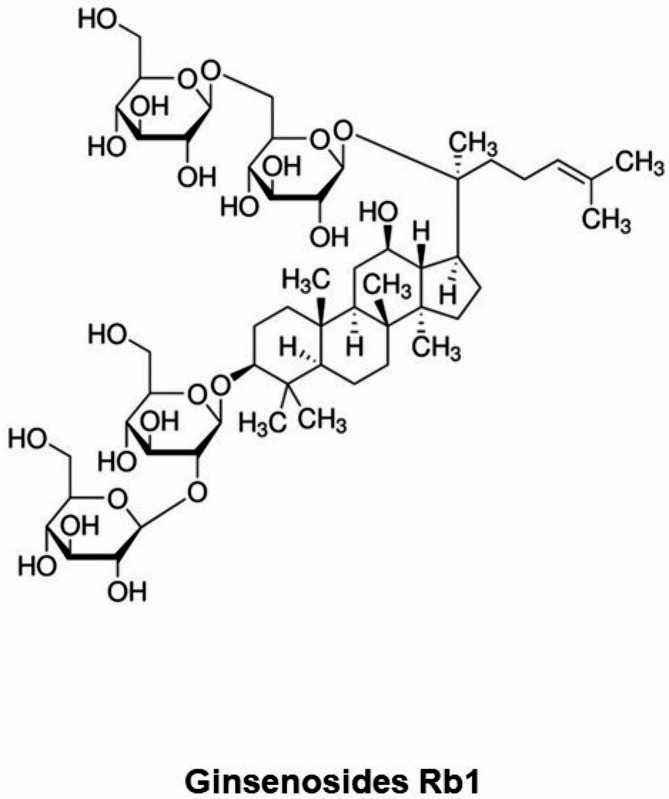
Types of ginsenosides Ginsenosides Rb1

[3H]Ryanodine (specific activity 87 Ci/mmol) was purchased from PerkinElmer Life Science Products (Boston, MA). RyR2 antibodies (MA3-925) and phospho-RyR2 (Ser2808) polyclonal antibodies (PA5-105712) were obtained from Thermo Fisher Scientific. Streptozotocin (STZ) was obtained from Sigma-Aldrich (St. Louis, MO). Ginsenosides Rb1 (CAS number: 41753-43-9) were purchased from Shanghai Tauto Biotech Co., Ltd. (Shanghai, P.R. China); The molecular construction of Rb1 is shown in Fig. 1. Other reagents and solvents used were of analytical grade and were purchased from Sigma-Aldrich (St. Louis, MO).

### Animal preparations

Male Sprague-Dawley rats (6 weeks, weighing 200 ± 20 g, Beijing Vital River Laboratory Animal Technology Co., Ltd. Beijing, China) were used in this study, and the animal procedures used were approved by the Institutional Animal Care and Use Committee of China Academy of Chinese Medical Sciences Guanganmen Hospital (case number GAM/EC09/2019). Rats were housed in conditions with a temperature of 22 ± 2 °C and a 12:12-h light/dark cycle, with food and water.

### STZ induced type 1 diabetes

STZ-induced type 1 diabetes was performed as in a previous study [15]. In brief, 30 rats were injected with 45 mg/kg of streptozotocin solution (STZ), which formulated with a 1% solution of 0.1 mol/L of citric acid buffer in an ice bath (pH = 4.4), and another 10 rats were injected with citrate buffer only to serve as controls. Two weeks after STZ injection, blood glucose was measured using tail clipping method in each rat one week later, and a blood glucose value > 16.7 mmol/L was considered to be indicative of successful replication of the diabetic rat model.

### Treatment of diabetic animals

**Fig. 2:**
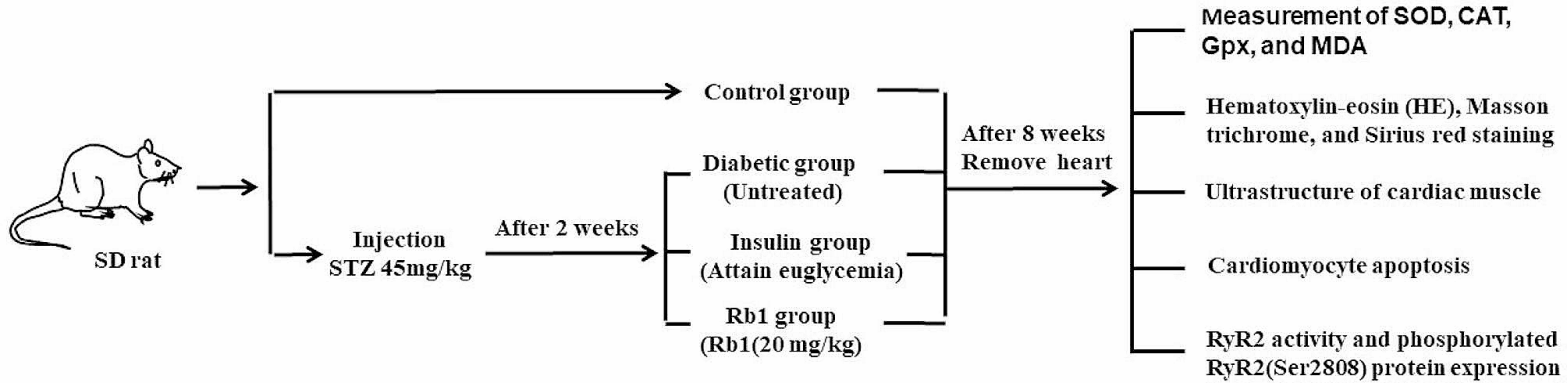
A schematic of the study design

Two weeks after STZ injection, 30 diabetic model rats were randomly divided into the following groups: the diabetic group (*n* = 10) received no treatment; the insulin-treated diabetic group (insulin, *n* = 10) was treated with insulin (15 U/kg, sc) for 8 weeks; and the Rb1 treated diabetic group (Rb1, *n* = 10) received intragastric Rb1 (20 mg/kg of Rb1) for 8 weeks [24, 26]. A schematic of the study design is shown in Fig. 2.

### Echocardiography

Echocardiography was performed as previous study [29]. Animals were in lightly anesthetized (0.3 mL of a cocktail containing 100 mg/ml ketamine and 10 mg/mL acepromazine given i.p.). M-mode echocardiography was performed at the end of the 8-week protocol using high resolution echocardiography system at 8 weeks. Left ventricular end-diastolic diameter (LVEDD), end-systolic diameter (LVESD), left ventricular end-diastolic volume (LVEDV) and end-systolic volume (LVESV) were measured parameters. Percent fractional shortening (FS) = [(LVEDD - LVESD)/LVEDD] x 100. Percent ejection fraction (EF) = [(LVEDV - LVESV)/LVEDV] x 100.

### Collection of tissue samples

At 8 weeks of treatment, after their blood glucose levels and weight were tested, rats were anesthetized using a vaporizer (5% isoflurane) through a nose cone. Once a deep plane of anesthesia has been reached, their thoracic cavities were opened, the hearts were rapidly removed and placed in a cold isotonic PBS buffer, dried with filter paper. The left ventricle was cut into 3 parts: one part was immersed in paraformaldehyde at 4^∘^C for 24 h, and the other parts were placed in liquid nitrogen until use.

### Assays of antioxidant activity and lipid peroxidation

Cardiac tissue was homogenized with KCl at a 1:10 ratio. The homogenate was centrifuged (9000 x g, 30 min), and the supernatant was used for the measurement of SOD, CAT, Gpx, and MDA with commercially available assay kits (SOD, A001-3; CAT, A007-1-1; Gpx, A005-1-1; and MDA, A003-1; Jiancheng Biotech, Nanjing, China) according to the manufacturer’s protocols. Total.

protein in each sample was measured according to the Bradford protein assay [26].

### Preparation of RyR2 vesicles

RyR2 membrane vesicles were prepared from rat hearts using procedures described previously [30]. Briefly, Heart muscle (50 g) was minced and homogenized (5 × 20 s, speed setting 4.5,

ProScientific, Oxford, CT, USA) in 350 ml of 0.3 M sucrose, imidazole·HCl 10mM, phenylmethylsulfonyl fluoride (PMSF) 230µM, leupeptin 1.1µM, pH 7.4 at 4^o^C for 1 min. The homogenate was centrifuged for 20 min at 7,500 gav (average g) for 20 min. The pellet was resuspended with isolation buffer and homogenized 10 s, and centrifuged at 11,000 gav for 20 min. The supernatant was centrifuged at 45,000 gav for 30 min, The crude RyR2 vesicles pellets were collected and saved at -80oC.

### RyR2 activity

Ryanodine binds with high specificity to the RyRs and is widely used to determine sensitivity of RyR2 to Ca2 + activation. In this study, the RyR2 activity was determined as per the procedures described previously [30, 31]. Briefly, crude RyR2 Vesicles (0.1 mg/ml) were incubated in binding buffer including 500 mM KCl, 20 mM Tris-HCl, 0.3 mM Ca^2+^, 2 mM reduced glutathione, 0.1 mM EGTA, and 6.7 nM [3H]ryanodine. After incubation for 2 h at 37 °C. At the end of the incubation, samples were rapidly filtered through GF/C filters, washed three times with ice-cold binding buffer, the samples were filtered and washed, and the amount of [3H]ryanodine bound was determined using liquid scintillation counting.

### Determination of phosphorylated RyR2(Ser2808)

Phosphorylated RyR2(Ser2808) is protein kinase A (PKA)-mediated phosphorylation of the cardiac RyR2. Hyperphosphorylation of RyR2-S2808 have been indicated as a mechanism contributing to arrhythmogenesis and heart failure. In this study, Western blot analysis of RyR2 and RyR2-S2808 was performed as described previously [31]. Briefly, 50 µg of RyR2 vesicles was run on a 4–15% gradient Tris–glycine polyacrylamide gels (BioRad Technologies Inc, Burlingame, CA) for 210 min at 150 V, and then transferred to polyvinylidene difluoride membranes (PVDF, Millipore Corporation, Bedford, MA) overnight at 4 °C. Primary antibody was incubated at either 4 °C (overnight) or room temperature (3H). Primary antibodies used: mouse-anti-RyR2 (1:1500), rabbit-anti-RyR2-pS2808 (1:1000), and mouse-anti-β-actin (1:1000). Blot Imager with analysis performed in ImageJ software.

### Histopathology

Hematoxylin-eosin (HE), Masson trichrome, and Sirius red staining are the mainstay of diagnostic histopathology, which conveniently highlight the structural and pathological changes, amount and distribution of fibrosis. Histopathology of heart tissue was performed as described previously [32]. Briefly, myocardial samples were cut transverse 8.0-µm thick serial sections (via a rotary microtome (Leitz, 1512, Germany) from paraffin embedded left ventricle tissue slices. Hematoxylin-eosin (HE), Masson trichrome, and Sirius red staining were performed according to the manufacturer’s instructions. The sections were finally observed and imaged under a light microscope, and quantified with Image J software (NIH).

### Transmission electron microscopy (TEM)

TEM sample preparation was performed as described previously [33]. Briefly, small specimens of left ventricular tissue from each group of rats were collected and washed three times in cold PBS (10 min each). The tissues undergo submersion fixation in 1% osmic acid in 0.1 M Na cacodylate buffer for 2 h at room temperature and are soaked in epoxy resin for 2 h before embedding. The resin-embedded tissues were placed in an incubator with a thermostat for 24 h of polymerization. The ultra-fine sectioning is performed using an ultramicrotome, where sections of 1 μm are cut. The ultrathin sections were stained with uranyl acetate and lead citrate before rinsing three times. The ultrastructure of the tissue from each group was observed with a JOEL.

100 II transmission electron microscope (Massachusetts, Boston, USA).

### Tunel assay

Terminal deoxynucleotidyl transferase (TdT) dUTP Nick-End Labeling (TUNEL) assay identify apoptotic cells by DNA fragmentation occurs during the late stages of apoptosis. In this study, the tunel assay was performed as described previously [34, 35]. Briefly, formalin-fixed sections of cardiac tissue slides were treated with H2O2 and then with fluorecein-12-dUTP (50 μm) in the presence of dATP (100 µM) and TdT enzyme at 37 °C. The samples were incubated with DAPI for 2 min at room temperature and placed at 4 °C on a coverslip. The section was observed with light before microscopy.

### Data analysis and statistics

Statistical analysis was performed using analysis of variance employing SPSS 22.0 software.

(IBM SPSS Inc., Chicago, IL). The data were analyzed by one-way ANOVA, followed by Bonferroni’s multiple comparison test. The data are presented as the mean ± SEM. In all cases, the level of statistical significance was set at *P* < 0.05.

## Results

### General characteristics and hemodynamic parameters

The blood glucose levels of the control, STZ diabetic, insulin, and Rb1 groups were 4.07 ± 0.08,

22.69 ± 1.34, 5.27 ± 0.09, and 18.62 ± 2.44, respectively. The STZ diabetic group had a significantly higher blood glucose level than the control group (*P* < 0.01), confirming the successful modeling of diabetes. The blood glucose level of the Rb1 group was significantly higher than that of the control group (*P* < 0.01), and that of the insulin group was also significantly lower than that of the diabetic group (*P* < 0.01); The data on the blood glucose levels of the control and insulin groups showed no statistically significant difference. Diabetes, insulin, and Rb1 groups significantly lowered body mass and serum insulin levels (*P* < 0.01) from control, and the insulin group increased body mass and serum insulin levels (*P* < 0.05). The Rb1 group increased body mass but did not significantly alter serum insulin levels compared to the diabetic group (Table 1).

**Table 1:** General characteristics of animals used in the study

Parameter	Control *n* = 10	Diabetic *n* = 10	Insulin *n* = 10	Rb1 *n* = 10
Body mass(g)	401 ± 4.28	289.5 ± 20.18**	342.17 ± 11.58**^#^	343.67 ± 13.5**^#^
Heart weight(g)	1.41 ± 0.06	1.11 ± 0.03*	1.36 ± 0.05^#^	1.33 ± 0.05^#^
Blood glucose (mM)	4.07 ± 0.08	22.69 ± 1.34**	11.08 ± 1.64**##	18.62 ± 2.44**
Serum insulin (ng/ml)	21.01 ± 0.63	12.64 ± 0.54**	16.38 ± 0.68**##	14.62 ± 1.36**

* *P* < 0.05 and ** *P* < 0.01 from control; # *P* < 0.05 from diabetic, ## *P* < 0.01 from diabetic

### M-Mode echocardiography

**Fig. 3:**
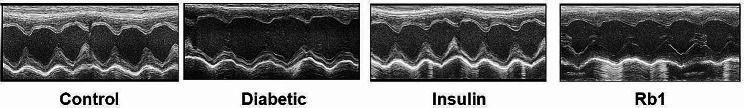
Representative echocardiograms of control, diabetic, insulin, and Rb1

After 8 weeks of diabetes, FS% were significantly (*p* < 0.01) reduced in diabetic rats compared with controls, FS% of insulin and Rb1 group significantly (*p* < 0.05) increased compared with diabetes (*P* < 0.05); EF% were significantly reduced in diabetic compared with controls (*p* < 0.01), insulin and Rb1 group significantly increased compared with diabetes (*P* < 0.01, *P* < 0.05, respectively); There was not significantly differently alter among of control, insulin, and Rb1 group in both FS% and EF% (Table 2; Fig. 3).

**Table 2:** The parameters of echocardiography

Parameter	Control *n* = 6	Diabetic *n* = 6	Insulin *n* = 6	Rb1 *n* = 6
FS	60.25 ± 1.32	48.94 ± 2.49**	59.28 ± 1.24^#^	57.84 ± 2.73^#^
EF	78.15 ± 1.44	64.11 ± 0.7**	73.62 ± 1.57^##^	71.59 ± 2.77^#^

FS: % Cardiac fractional shortening, EF: % Cardiac ejection fraction; ** *P* < 0.01 from control; # *P* < 0.05 and ## *P* < 0.01 from diabetic

### SOD, CAT and Gpx activity and level of MDA in heart tissue

**Fig. 4:**
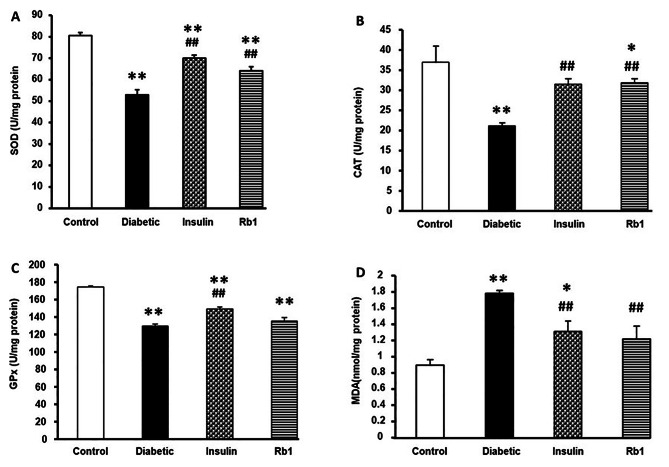
SOD, CAT, Gpx activity, and MDA level in the heart tissue (**A**) SOD, (**B**) CAT, (**C**) Gpx activity, and (**D**) MDA level in heart tissue of control, diabetic, insulin, and Rb1 rats. The bars stand for means ± S.E.M. * *P* < 0.05 and ** *P* < 0.01 in control rats; ## *P* < 0.01 in diabetic rats by one-way ANOVA

The activities of SOD, Gpx, CAT, and level of MDA in heart tissue are shown in Fig. 2. SOD activity significantly decreased in the diabetes (53.05 ± 2.32), insulin (70.2 ± 1.22), and Rb1 (64.38 ± 1.67) groups from the control group (80.69 ± 1.26) (*P* < 0.01); SOD activity significantly increased in the insulin and Rb1 groups from the diabetic group (*P* < 0.01) in Fig. 4A. CAT activity significantly decreased in the diabetes group (22.39 ± 0.65, *P* < 0.01) and the Rb1 group (24.83 ± 0.41, *P* < 0.05) from the control group (36.95 ± 4.09); the insulin group (27.79 ± 0.53) was not significantly different from the control group; and the insulin and Rb1 groups in CAT activity significantly decreased from the diabetes group (*P* < 0.01) in Fig. 4B. Gpx activity significantly decreased in the diabetes (129.72 ± 2.38, *P* < 0.01), insulin (149.57 ± 2.1, *P* < 0.01), and Rb1 (135.6 ± 4.21, *P* < 0.01) groups from the control group (174.81 ± 0.92); insulin in Gpx activity significantly increased in the diabetes group, but the Rb1 group was not significantly different from the diabetic group in Fig. 4C. The level of MDA in the heart significantly increased in the diabetes (1.79 ± 0.03, *P* < 0.01), and insulin (1.32 ± 0.13, *P* < 0.05) groups from the control group (0.89 ± 0.07); the Rb1 group (1.22 ± 0.16) was not significantly altered from the control group; and the insulin and Rb1 groups levels of MDA significantly decreased from the diabetes group (*P* < 0.01) in Fig. 4D.

### Histopathology

**Fig. 5:**
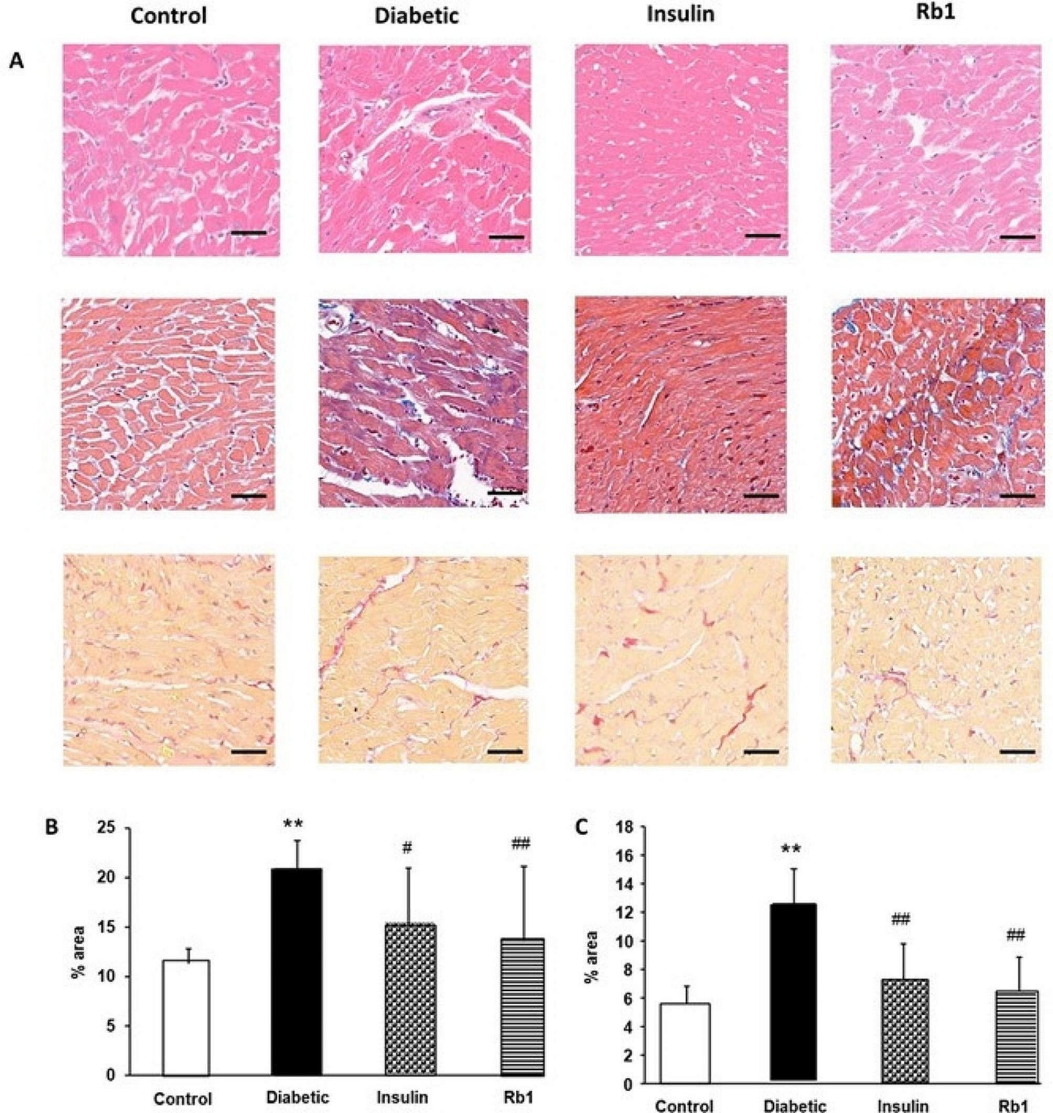
Morphological changes of heart tissue in diabetes mellitus. (**A**) Control, diabetic, insulin, and Rb1 rat myocardial tissue were stained with HE, Masson trichrome, and Sirius red staining. The diabetic cardiac muscle fibers were disordered. Fibrosis in diabetic cardiomyocytes was more attenuated with insulin and Rb1 treatment. Bar = 50 μm. (**B**) quantitative analysis of the internal fibrosis from masson staining; (**C**) quantitative analysis of the internal fibrosis from Sirius red staining

HE, Masson’s trichrome, and sirius red staining of cardiac tissues revealed clear structural abnormalities. HE staining of cardiac tissues showed an irregular shape, cell hypertrophy, an increased cell gap, myocardial fiber disarrangement, muscle fiber fracture, and increased extracellular matrix deposition in the DCM group compared with the normal control group (Fig. 5A). The amount of collagen accumulation in myocardium was significantly increased in rats from the DCM group, and the extent of fibrosis seemed to be similar to controls in the insulin and Rb1 treatment diabetic group. Histological analysis indicated that myocardial hypertrophy, the fibrosis area, and extracellular matrix deposition in the heart in the diabetic group were improved after insulin and Rb1 treatment (Fig. 5B and C).

### TEM findings

**Fig. 6:**
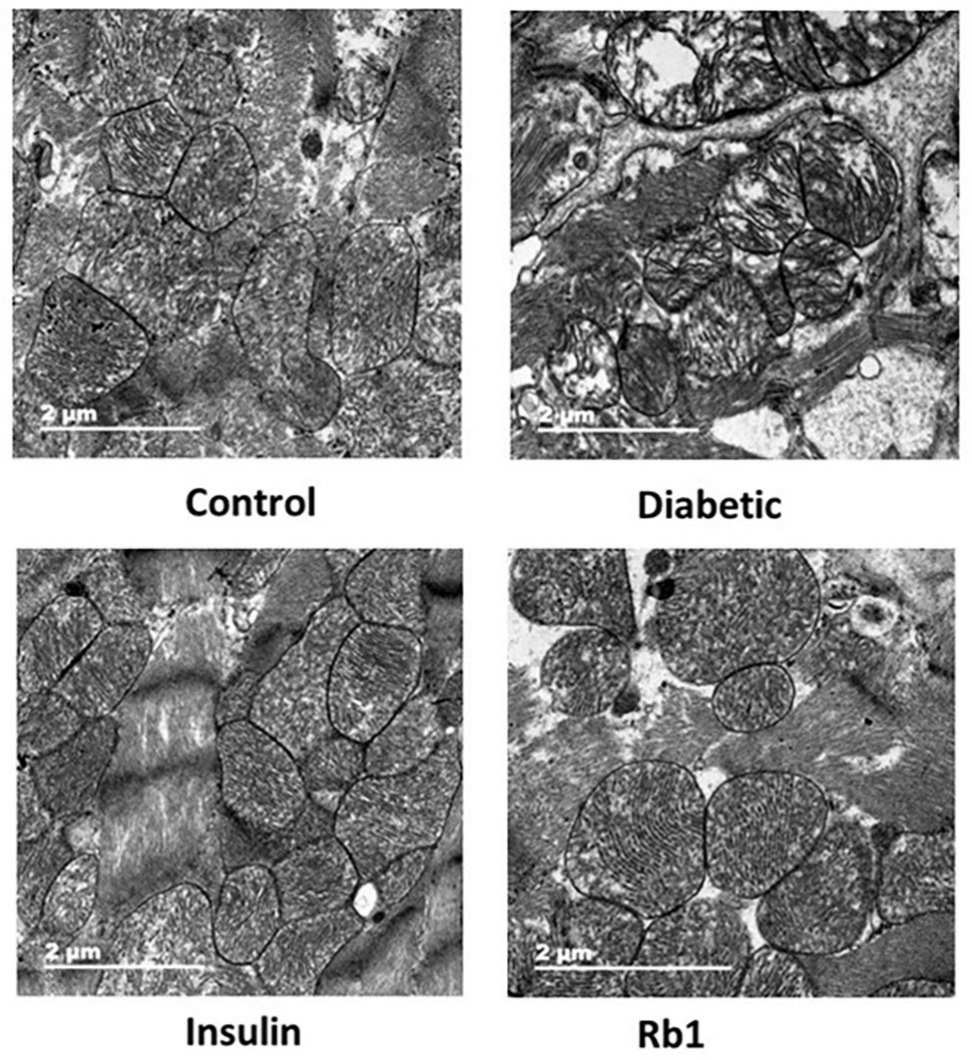
Analysis of cardiac ultrastructure. Representative transmission electron microscopy images showed that mitochondrial cristae morphology changed in diabetic rats. Rb1 treatment alleviates the damaged mitochondria. Bar = 2 μm

Mitochondrial morphology and content were observed by electron microscopy (Fig. 6). The ultrastructure of mitochondria showed normal outer and inner mitochondrial membranes in control rats. The normal mitochondria had tightly packed cristae with parallel alignment and a higher matrix density. Mitochondria in cardiac muscle cells showed dysorganized cristae and reduced cristae density in diabetic rats. Mitochondria showed morphological alterations in insulin- and Rb1-treated rats.

### Tunel positive cardiomyocytes

**Fig. 7:**
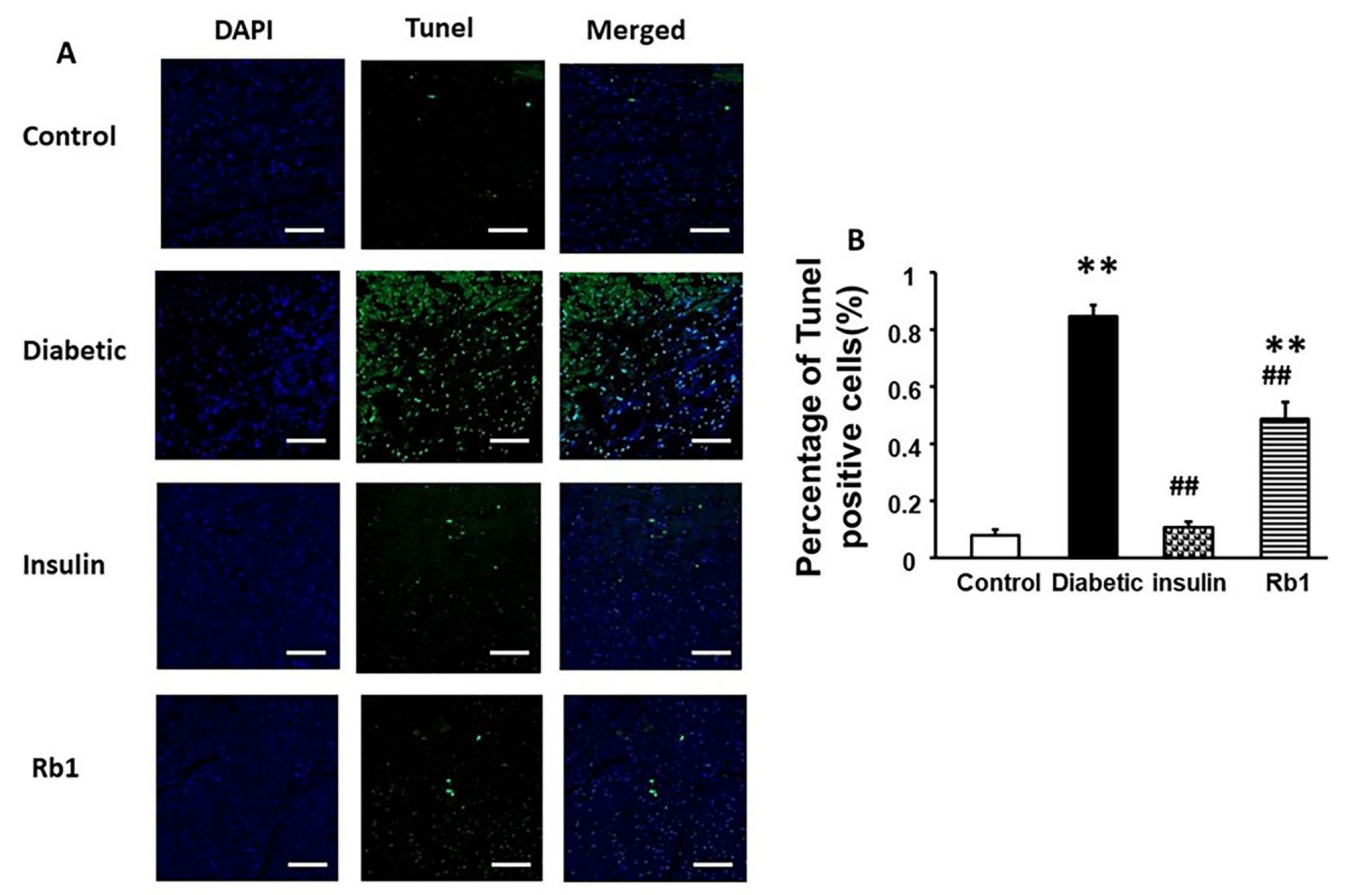
Rb1 reduces cardiomyocyte apoptosis in diabetic rats. (**A**) Representative pictures of myocardial tissue sections stained with TUNEL bar = 25 μm. (**B**) Data shown as Means ± SEM **p < 0.01 from the control group, and ##*p* < 0.01 from the diabetic group by one-way ANOVA

TUNEL-positive cells were evaluated by the TUNEL staining assay (Fig. 7). 84.79 ± 3.9% of TUNEL-positive cells in the diabetic group were higher than those in the control group (8.19 ± 1.76%) (*p* < 0.01). Treatment with insulin and Rb1 significantly decreased the percentage of apoptotic myocardial cells (insulin, 10.77 ± 1.99%, and Rb1, 48.74 ± 5.89%; *p* < 0.01). These results indicate increased apoptosis of the myocardium in the diabetic group, and Rb1 attenuates cardiomyocyte apoptosis in diabetic rats.

### RyR2 activity and relative levels of RyR2 phosphorylation at Ser2808

**Fig. 8:**
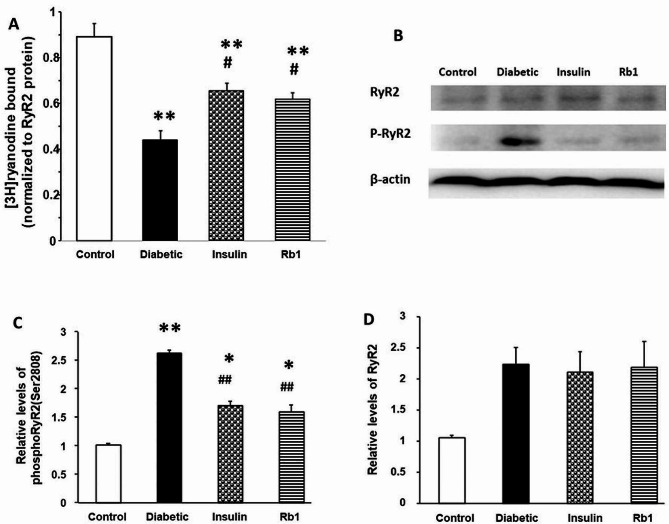
[3H]ryanodine-binding activity and levels of RyR2 in control, diabetic, insulin, and Rb1 rats. (**A**) Comparison of the amount of [3H]ryanodine capable of binding to membrane vesicles from control, diabetic, insulin, and Rb1 rat hearts. The bars stand for means ± S.E.M. (**B**) Representative western blots of total RyR2 and phosphor-RyR2 (Ser2808) levels (**C, D**). The graph at the bottom shows the mean ± SE of relative RyR2 and pRyR2 (Ser2808) levels. ** *P* < 0.01 from control rats; # *P* < 0.05; and ## *P* < 0.01 from diabetic rats by one-way ANOVA

Neutralization of RyR2 activity was significantly decreased in diabetes (0.44 ± 0.04, *P* < 0.01), insulin (0.65 ± 0.03, *P* < 0.01), and Rb1 (0.61 ± 0.03, *P* < 0.01) groups from the control group (0.89 ± 0.06); insulin group (*P* < 0.05) and Rb1 group (*P* < 0.05) were significantly increased from the control group (Fig. 8A). One potential mechanism for increased sensitivity of RyR2 to Ca2 + activation is an increase in RyR2 and phosphorylation. In the present study, we found increased phosphorylation of RyR2 Ser2808 (1.6 folds over control) in the hearts of STZ-diabetic rats. Increases in phosphorylation of RyR2 at Ser2808 was attenuated by insulin and Rb1 treatment. We also found that despite the increasing level of RyR2 protein in the diabetic, insulin, and Rb1 groups, the RyR2 protein was not significantly different between the sample types of groups (Fig. 8B-D).

## Discussion

Diabetic cardiomyopathy has been recognized as an important cause of heart failure and mortality in diabetic populations, but there remains no effective medicine to prevent the development of the disease and mortality. In this study, our data demonstrated that the diabetics had decreased body weight, blood glucose level, and serum insulin level; insulin treatment had improved the levels of blood glucose, serum insulin level, and body weight. Rb1 improved the body weight of diabetic rats.

The principal finding of the present study is that carbonylation oxidative stress from hearts of STZ induced diabetic rats with established diabetic cardiomyopathy; Rb1 and insulin treatment increased SOD activity and reduced MDA adducts in cardiac muscle of STZ diabetic rats. Abnormal chemical reactions in hyperglycemia alter normal metabolic processes in diabetes, which is a key process in the production of ROS and RCS. Increasing the concentration of ROS and RCS may result in oxidative stress in the diabetic heart. Oxidative stress is associated with increased oxidative damage and cardiomyocyte death, which contributes to increased interstitial fibrosis [11]. Our data confirm that oxidative stress and fibrosis are also features of DCM and hypertension. Insulin and Rb1 increased SOD 1.32-fold and 1.21-fold, catalase enzymes 1.48-fold and 1.11-fold, Gpx 1.15-fold and 1.05-fold, and decreased MDA levels 1.34-fold and 1.46-fold in diabetic myocardial tissue, respectively. These data suggested that insulin and Rb1 reduced oxidative stress. The mechanisms by which insulin decreased oxidative stress could reduce hyperglycemia, which altered abnormal metabolic processes in diabetes. The possible mechanisms by which Rb1 reduced oxidative stress could be improved mitochondrial lipid metabolism, which produced ROS and RCS [36]. Numerous studies have shown that the progression of DCM is associated with chronic inflammation and cardiac cell death, which have been linked to the NLRP3 inflammasome [37, 38]. Glucose stimulates excessive ROS and RCS generation, which are known activators of NLRP3 inflammasomes [39]. This could be a critical mechanism triggering NLRP3 inflammasome formation and activation in response to Rb1, which alleviates oxidative stress and improves cardiac dysfunction.

The oxidative state may play a role in cardiac fibrosis and apoptosis related to DCM [40].

Mitochondria are essential organelles for the generation of ROS. Oxidative stress could lead to.

DNA damage and fibrosis, mitochondrial dysfunction, and mitochondrial damage results in cardiomyocytes death. This is consistent with other studies [41, 42]. In this study, histological stains showed that the insulin and Rb1 groups had fewer myocardial cells and less muscle fiber disarrangement compared with the diabetic group. It has been demonstrated by TEM that damaged mitochondria can affect the function of cardiomyocytes in the diabetic heart, and TUNEL-positive cardiomyocytes were increased in the myocardium of diabetic rats. Our study also showed that insulin and Rb1 treatment significantly decreased the percentage of apoptotic cardiomyocytes and improved most of the mitochondrial pathological changes in the myocardium of diabetic rats. This result is consistent with that of a previous study [34, 35]. This observation suggests that insulin and Rb1 ameliorate cardiac remodeling in diabetic rats, although their possible mechanisms of action are different.

Another major finding of the present study is that phosphorylation RyR2 at S2808 by carbonylation oxidative stress is important mechanisms of diabetic cardiomyopathy; Rb1 and insulin treatment is more efficacious way to reduce the process of phosphorylation RyR2. Post-translational modification by RCS and oxidative stress contribute to the heterogeneity in RyR2 activity deregulation [11, 30, 43, 44]. We also found that at equivalent amounts of RyR2 protein from diabetes rats, the heart bound less [3H] ryanodine activity compared with control rats [45]; insulin and Rb1 treatment increased the [3H] ryanodine activity. The hyperglycemic body alters normal metabolic processes, which are also critical in the production of ROS and RCS. This increased concentration of ROS and RCS may result in the phosphorylation of RyR2 in diabetes [11]. Phosphorylation at S2808 can enhance the luminal (intra-SR) sensitivity of RyR2, leading to increases in RyR2 open probability and promotion of diastolic Ca2 + leak [46]. Previous studies have described an increase in RyR2 phosphorylation at the Ser2808 site in diabetic rats [36]. To address this, we used phosphospecific antibodies to assess the phosphorylation of RyR2 at Ser2808. We found that although total RyR2 remained significantly unchanged, phosphorylation of RyR2 at Ser2808 increased significantly during diabetes [47], and these increases were attenuated by insulin and Rb1 treatment. These data directly show that Rb1 attenuates increases in RyR2 phosphorylation at Ser2808 in diabetic rats.

Limitations of this trial include maybe have difference between fresh and frozen heart tissue; fresh heart tissue should be better as fixation is a complex series of chemical events; the experiments was challenging as a result of using fresh heart tissue because of the long-term experiment.

## Conclusion

Our study provides strong evidence in support of the increased ROS, RCS, and RyR2 phosphorylation levels in myocardium contributes to the pathogenesis of diabetic cardiomyopathy. We demonstrated a novel finding that Rb1 improved body weight in diabetic rats and degraded myocardial interstitial fibrosis. The mechanism for Rb1 protecting the myocardia of diabetic rats is reducing or scavenging ROS, RCS production, and RyR2 phosphorylation in diabetes mellitus. Moreover, the present study establishes that the mechanism of Rb1 in diabetic cardiomyopathy involves ROS, RCS, and RyR2. Decreased ROS and RCS generation and enhanced RyR2 binding activity may represent a method to treat cardiomyopathy, arrhythmias, and heart failure.

## Data Availability

The datasets used and analyzed during the current study are available from the corresponding author upon reasonable request.
